# Immune Response of Pigs Vaccinated Against Proliferative Enteropathy and Co-Infected with *Lawsonia intracellularis* and *Brachyspira hyodysenteriae*

**DOI:** 10.3390/ani16010114

**Published:** 2025-12-31

**Authors:** Sarah Chagas, Peyton Jensen, Eliana Paladino, Lívia Mendonça Pascoal, Stephan von Berg, Connie Gebhart, Fabio A. Vannucci

**Affiliations:** 1 College of Veterinary Medicine, University of Minnesota, St. Paul, MN 55108, USA; 2 Escola de Veterinária e Zootecnia, Universidade Federal de Goiás, Goiânia 74001-970, GO, Brazil; 3 MSD Animal Health, 85716 Munich, Germany

**Keywords:** cellular response, cytokine, humoral response, ileitis, swine dysentery

## Abstract

Vaccination is a tool to control *Lawsonia intracellularis* (LI) in pigs. However, pigs may have co-infections that worsen clinical signs and lesions. The aim of this study was to characterize systemic and gut-mediated humoral and cell-mediated immune (CMI) responses in pigs vaccinated with a killed intramuscular LI vaccine and to analyze the impact of co-infection with *Brachyspira hyodysenteriae* (Bhyo) on the immune response. The study included eighty pigs and five study groups: V-CO (LI-vaccinated and co-infected with LI + Bhyo, *n* = 21), P-CO (placebo and co-infected with LI + Bhyo, *n* = 18), V-LI (LI-vaccinated and infected with LI, *n* = 21), P-LI (placebo and infected with LI, *n* = 12), and NC (negative control, placebo and non-challenged, *n* = 8). Parameters analyzed: fecal score and pathogen shedding), gross intestinal lesions, LI intestinal colonization (IHC), serum IgG, LI-specific IFN-γ production (ELISPOT), and immune cell subsets (flow cytometry) in blood, mesenteric lymph nodes, Peyer’s patches, and intestinal epithelium. LI vaccination significantly reduced LI fecal shedding, intestinal colonization, and macroscopic lesions—even under Bhyo co-infection. Vaccinated pigs had earlier and stronger serum IgG and IFN-γ responses. B cells seem to play an important role in the local immune response, and T regulatory cells apparently do not have a significant role in immunomodulation. This study contributes to a better understanding of LI immune response and can provide subtract for further research in the control of LI.

## Simple Summary

*Lawsonia intracellularis* is a bacterium that causes intestinal disease in pigs, leading to poor growth and economic losses. Vaccination is one of the main ways to control this infection, but pigs are often exposed to other pathogens such as *Brachyspira hyodysenteriae*, which can worsen the disease. In this study, we evaluated how the immune system of pigs reacts to a vaccine made from inactivated *L. intracellularis* and how co-infection with *B. hyodysenteriae* affects this response. We examined several aspects of the pigs’ health, including intestinal lesions, presence of bacteria in the feces, and immune activity in the blood and intestine. The vaccinated pigs had fewer intestinal lesions, lower bacterial shedding, and stronger immune reactions than unvaccinated animals, even when exposed to both bacteria. These findings show that vaccination helps pigs fight against *L. intracellularis* infection and remain healthier, and they also provide new insights into how the immune system protects the gut. This information can help scientists and veterinarians develop better strategies to control intestinal diseases in pig production.

## 1. Introduction

*Lawsonia intracellularis* (LI) is a pathogenic intracellular bacterium that causes proliferative enteropathy (PE) in pigs [1]. PE is endemic in all major pork-producing countries [2,3,4,5] and causes significant economic losses annually. Commercial vaccines have been shown to improve performance, reduce clinical signs, and enteric lesions; however, they do not prevent infection and transmission or eliminate fecal shedding [6,7,8,9,10].

Co-infection with other pathogens can aggravate LI infection. Multiple microorganisms co-exist in the intestine and can interact and synergism between LI and *Brachyspira hyodysenteriae* (Bhyo) has been reported [11,12,13]. Bhyo causes swine dysentery (SD) and the impact of this bacterial interaction/synergism on the LI immune response remains unclear.

There are still gaps in understanding the immune response to LI infection. Regarding the humoral response, serum-specific IgM is present during active disease [14]; serological IgG is detected around 2 weeks and peaks around 3 weeks after challenge [1]. Mucosal IgA can also be identified in the intestinal lavage 3 weeks after infection [15].

Given the intracellular nature of LI, cell-mediated immune (CMI) responses are expected to play an important role in protective immunity. Typical PE lesions have a limited inflammatory component, suggesting immunomodulation by LI. Macintyre et al. (2003) [16] demonstrated a reduction in T and B cells in LI infection; studies have analyzed systemic CMI response [6,8,17,18] and have found IFN-γ production in pigs after LI challenge. IFN-γ has shown an important role in the protection against LI [15,19] by limiting the development of lesions [20]. However, there is still a lack of knowledge, especially in the post-vaccination immune response in pigs immunized with vaccines against LI.

The aim of this study was to characterize systemic and gut-mediated humoral and CMI responses in pigs vaccinated with a killed intramuscular LI vaccine and to analyze the impact of co-infection with Bhyo on the immune response.

## 2. Materials and Methods

### 2.1. Animals and Study Design

Eighty pigs at the age of 21 days derived from an LI-free farm (Fergus Falls, MN, USA) were obtained for this study. Serum samples were collected from the sows that farrowed and nursed the piglets, and these samples were sent to the Veterinary Diagnostic Laboratory at the University of Minnesota (VDL-UMN) for LI serological testing. All sows were negative for the presence of LI-IgG in their serum. The trial was approved by the Institutional Animal Care and Use Committee (IACUC) of the University of Minnesota.

**Figure 1:**
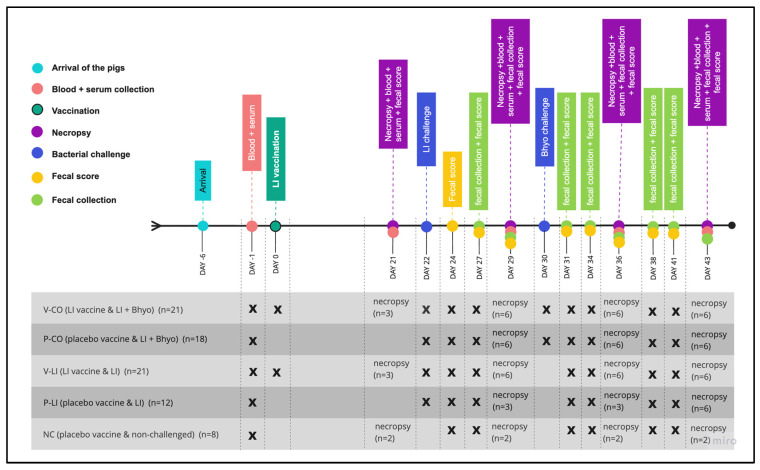
Study design is represented by a timeline of the main events and the experimental groups. LI = Lawsonia intracellularis, Bhyo = Brachyspira hyodysenteriae, V-CO = LI-vaccinated and co-infected with LI + Bhyo group, P-CO = placebo and co-infected with LI + Bhyo group, V-LI = LI-vaccinated and infected with LI group, P-LI = placebo and infected with LI group, and NC = negative control, placebo, and non-challenged group.

Pigs were housed in the isolation facility at the University of Minnesota. By the time the pigs arrived (D-6), they were weighed, divided into five groups, and randomized by weight: V-CO (LI-vaccinated and co-infected with LI + Bhyo, *n* = 21), P-CO (placebo and co-infected with LI + Bhyo, *n* = 18), V-LI (LI-vaccinated and infected with LI, *n* = 21), P-LI (placebo and infected with LI, *n* = 12), and NC (negative control, placebo, and non-challenged, *n* = 8) (Figure 1). Groups were housed in separate rooms with ad libitum feed and water. Pigs from each group were housed together in a single pen measuring 5.61 m^2^.

Pigs had five days for acclimatation and one day before vaccination (D-1). EDTA whole blood samples from the pigs from all groups were collected and tested for LI-specific IFN-γ production using an ELISPOT assay following the manufacturer’s recommendations (Porcine IFN-gamma ELISpot Kit EL985, R&D Systems, Minneapolis, MN, USA).

### 2.2. Vaccination

On day zero (D0), pigs in the V-CO and V-LI groups were intramuscularly vaccinated with 2 mL of killed LI vaccine (Porcilis Ileitis, serial number 92518127, Merck Animal Health, Madison, WI, USA). The vaccine was an oil-in-water emulsion, ready-to-use, and was stored in the refrigerator with a temperature ranging between 2 °C and 5 °C up to the moment it was used. Placebo groups (P-CO and P-LI) and the NC group received 2 mL of phosphate-buffered saline solution (PBS) intramuscularly (Gibco, Fisher Scientific, Waltham, MA, USA).

### 2.3. L. intracellularis and B. hyodysenteriae Challenge

All groups (V-CO, V-LI, P-CO, and P-LI) except NC were challenged with 50 mL of infected intestinal homogenate with 3.9 × 10^6^ organisms of LI per mL using an intragastric catheter, as previously described [8,17]. Pigs were challenged 22 days after vaccination (D22). The intestinal homogenate was purchased from a research company focused on diseases in swine (Gut Bugs, Inc., Fergus Falls, MN, USA); the company produced the inoculum [21] and froze it at 20 °C below zero; the inoculum was kept stored for 14 days up to the moment we thaw it to challenge the pigs. The lot number of the challenged homogenate used was GBI072122.

The V-CO and P-CO groups were also challenged with Bhyo pure culture (Bhyo 2019 field strain isolated from a diagnostic case from the VDL-UMN) eight days after LI challenge (D 30). A volume of 15 mL of the Bhyo pure culture with 1.4 × 10^8^ organisms of Bhyo per mL was orally administered to every pig within the previously mentioned groups. We used a lower Bhyo infectious dose than previously described [11,22], aiming to cause a subclinical disease in the pigs. To validate the subclinical Bhyo model, colonic intestinal content of the necropsied pigs after Bhyo challenge were collected and sent to the VDL-UMN to assess the positivity of the challenged pigs for Bhyo by real-time PCR (Table 2).

Bhyo was cultured in plates containing trypticase soy agar supplemented with 5% defibrinated sheep blood containing 12.5 mg/L of rifampicin, 200 mg/L of spectinomycin, 50 mg/L of vancomycin, and 12.5 mg/L of colistin under anaerobic conditions with gas mixtures N_2_ (80%), CO_2_ (10%), and H_2_ (10%) at 42 °C for 72 to 96 h [23].

### 2.4. Disease Monitoring

Pigs were monitored for general health condition and fecal score every day from D23 (one day post-LI challenge) to D42 (20 and 12 days post-LI and Bhyo challenge) (Figure 1). Following biosecurity procedures, all these parameters were monitored for the same evaluator.

The fecal score used ranged from 1 to 5 (1 = normal feces, 2 = moist feces, 3 = mild diarrhea, 4 = severe diarrhea, and 5 = watery diarrhea) [24]. All fecal swab samples were submitted to the VDL-UMN for LI and Bhyo real-time PCR (ThermoFisher TaqMan™ Fast Virus 1-Step Master Mix. Applied Biosystems, Waltham, MA, USA). The VDL-UMN uses its own protocol, validated for pig fecal and intestinal content samples.

Necropsies were performed on D21 (one day prior to LI challenge), D29 (7 days post-LI challenge and one day prior to Bhyo challenge), D36 (14 and 7 days post-LI and Bhyo challenge), and D43 (21 and 13 days post-LI and Bhyo challenge). The number of pigs necropsied per time point are described in Figure 1. The pigs designated for necropsy were randomly selected.

### 2.5. Tissue Sampling and Gross Pathology

Serum samples were collected on D21, D29, D36, and D43 for the LI Immunoperoxidase Monolayer Assay (IPMA), as previously described [17].

A fragment of Peyer’s patch (PP) (5 cm), mesenteric lymph node (LN) (1 lymph node), and a fragment of intestinal epithelium (5 cm) were collected and kept refrigerated in all pigs necropsied. Intestinal epithelium fragments were also collected to assess intraepithelial lymphocytes (IEL).

The blood, PP, LN, and IEL samples were processed for lymphocyte isolation and then analyzed by an ELISPOT assay for LI-specific IFN-γ (Porcine IFN-gamma ELISpot Kit EL985, R&D Systems, Minneapolis, MN, USA) and/or flow cytometry for non-specific cell-mediated immune response. The methods used for lymphocyte isolation, ELISPOT, and flow cytometry are described in Section 2.7.

The intestinal tract of euthanized pigs was examined for gross lesions characteristic of PE and SD. A five to eight cm fragment of the ileum closest to the ileocecal junction was collected in formalin 10% for LI immunohistochemistry (IHC). The method used for LI IHC is described below in Section 2.6.

### 2.6. Semi-Quantification of L. intracellularis Ileal Infection

All ileal fragments in formalin 10% were submitted to the VDL-UMN for *L. intracellularis* IHC, the gold standard laboratory diagnostic assay for LI. All fields in the histologic slide were observed. The slides were scored: 0—no staining, negative for LI; 1—focal presence of LI; 2—multifocal presence of LI; 3—diffuse presence of LI.

The IHC staining was performed in an autostainer (Autostainer Link 48, DakoCytomation, Copenhagen, Denmark) using a rabbit anti-LI primary polyclonal antibody [25] from personal laboratory stock. For quality control, the protocol included positive (a known LI-positive tissue slide incubated with the same anti-LI polyclonal antibody) and negative controls (a known LI-positive tissue slide incubated with negative control rabbit IgG fraction—DakoCytomation, Copenhagen, Denmark—in place of the anti-*Lawsonia intracellularis* antibody).

### 2.7. Cell-Mediated Immune Response

The Peripheral Blood Mononuclear Cell (PBMC) isolation method was performed using density gradient centrifugation [26]. Lymphocytes from LNs, IEL, and PPs were isolated [27,28]. A sample of 100 μL was taken from the isolated cells for count using an automatic cell counter (Cellometer K2 Fluorescent Cell Counter, Nexcelom Bioscience, Lawrence, KS, USA) using trypan blue dilution 1:2 (Gibco, Fisher Scientific, Waltham, MA, USA). We counted the live isolated cells to calculate cell concentration.

For the ELISPOT assay, 96-well plates from the Porcine IFN-γ ELISpot Kit EL985 (R&D Systems, Minneapolis, MN, USA) were used. For each pig sample, four wells were used with the following antigens: (1) sonicated LI antigen at 20 μg/mL, (2) sonicated McCoy cells at 8 μg/mL, (3) Concanavalin A (ConA) (Sigma-Aldrich, Saint Louis, MO, USA) at 10 μg/mL, and (4) no antigen.

Con A is used as a positive control, since it strongly stimulates lymphocytes to produce IFN-γ. McCoy cells are used as an antigen because the LI antigen is not pure—even after filtration and sonication; McCoy cells are still present on the LI antigen. McCoy cells can generate some unspecific reactions, so we established a threshold for what would be considered reactions specific for LI.

A total of 50 μL of the isolated lymphocytes at 10^7^ live cells/mL were added to each well (5 × 10^5^ live cells/well), except for the ConA well, which received 25 μL of the lymphocytes at 10^7^ live cells/mL (2.5 × 10^5^ live cells/well). Samples were tested in duplicate. The plates were incubated, washed, biotinylated, and stained following the recommendations of the ELISpot kit’s manufacturer.

For the flow cytometry, a panel of antibodies was used to characterize the cell subtypes. The antibodies and the isotype control antibodies used for immunostaining are listed in Table 1. Data were acquired using a FACSCanto™ flow cytometer (BD Biosciences, San Jose, CA, USA) and analyzed using FlowJo™ v10.10.

### 2.8. Statistical Analysis

For the fecal score, the One-Way Analysis of Variance (ANOVA) test was used to compare groups. For LI intestinal infection (IHC), the Kruskal–Wallis test was used to compare groups at every time point. For LI fecal shedding, IgG titration, and IFN-γ production, ANOVA was used for parametric data or a Kruskal–Wallis test was used for non-parametric data. For Bhyo fecal shedding, Student’s *t* test was used. For flow cytometry data, the frequencies of live cells in treatment groups were compared to the negative control (NC) group at each time point using ANOVA. A *p*-value of less than 0.05 was considered significant (*p* < 0.05). All the statistical analyses were performed using GraphPad Prism, version 10.2.0 (335) (GraphPad Software, San Diego, CA, USA).

## 3. Results

The IHC and PCR results successfully demonstrated an experimental infection for LI and Bhyo. A lower dose of Bhyo aimed to enable co-infection with LI (Table 2). This approach resulted in higher CT values in colonic samples, indicating a mild infection with Bhyo. Only one animal exhibited excessive mucus in the colon—a lesion typically associated with clinical Bhyo infection. This validates the intended co-infection model of clinical LI with subclinical Bhyo and provides a solid experimental foundation that reflects field conditions.

### 3.1. Disease Monitoring

The average fecal scores and standard deviation (StD) of all groups were analyzed and there were no significant differences between groups. V-CO had average score of 2.55 (StD = 0.8), P-CO 2.96 (StD = 1.01), V-LI 2.14 (StD = 1.02), P-LI 2.21 (StD = 1), and NC 1.05 (StD = 0.11). The individual fecal scores are reported in the Supplementary Material (Table S1).

Regarding LI fecal shedding, P-CO showed a lower average CT value (higher LI fecal shedding) compared to V-LI and P-LI on D31 (9 and 1 days post-LI challenge and Bhyo challenge) and D36 (14 and 6 days post-LI challenge and Bhyo challenge) (Table 3). No differences in Bhyo fecal shedding were observed between the LI-vaccinated (V-LI and V-CO) and placebo groups (P-LI and P-CO) (Table 4). The detailed CT values for LI are provided in the Supplementary Material (Table S2).

### 3.2. Gross Intestinal Lesions

**Figure 2:**
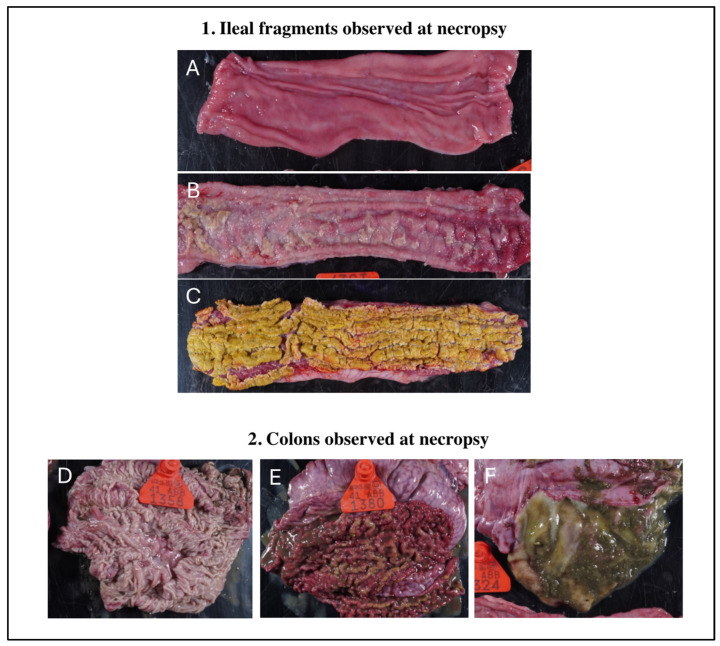
Board showing macroscopic lesions. Macroscopic photos were taken on D43, which is 21 dpi for *LI* and 13 dpi for *Bhyo*. 1. Gross lesions on ileal fragments: (**A**) normal ileal mucosa (NC pig). (**B**) Ileal lesions—edema, hyperemia, and thickening of mucosa (P-CO pig). (**C**) Ileal lesions—severe thickening of mucosa and necrosis (P-CO pig). 2. Gross lesions on colons: (**D**) a normal colon mucosa (P-CO pig). (**E**) Colon lesions—hyperemia, severe thickening of the mucosa, attached intestinal content, and diphtheritic membrane (P-CO pig). (**F**) Colon lesions—thickening of the mucosa and excessive mucus content (V-CO pig).

Gross intestinal lesions were only observed at 21 days post-LI infection and 14 days post-Bhyo infection. V-LI had no gross intestinal lesions and one out of six V-CO animals had gross lesions on the colon, typically caused by Bhyo. The placebo groups (P-LI and P-CO) had lesions on the ileum and colon, with three out of six animals in P-CO and two out of six in P-LI having intestinal gross lesions (Figure 2). The descriptions of the gross lesions observed in each pig are available in the Supplementary Material (Table S3).

### 3.3. L. intracellularis Intestinal Colonization

**Figure 3:**
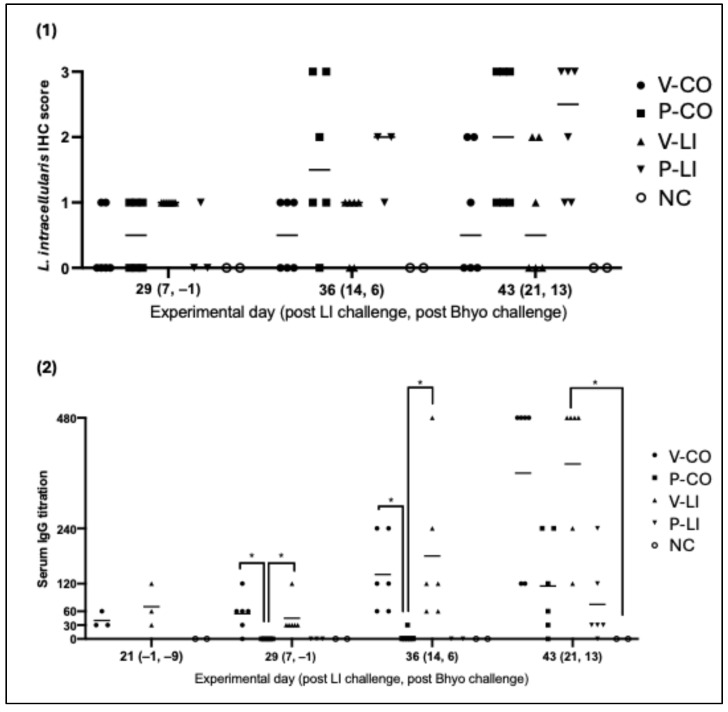
Board with plots. (**1**) Immunohistochemistry (IHC) scores for LI per group and at 3 different time points. IHC score was graded from 0 to 3—score 0 indicates no staining, score 1 indicates focal presence of LI, score 2 indicates multifocal presence of LI, and score 3 indicates diffuse presence of LI. Each dot represents one animal. (**2**) Anti-LI IgG titers on serum samples per group at 4 time points. Each dot represents one animal. Horizontal bars represent the average IgG titer of each group. Significant differences are represented as *p* < 0.05 (*).

Only the placebo groups (P-CO, P-LI) had a score of three (diffuse LI intestinal colonization) at the IHC evaluation—which corresponds to the highest level on the IHC score. No statistical difference was observed between groups, but by analyzing the data throughout the time, the ileal colonization in LI-vaccinated groups (V-CO and V-LI) lightly increased and no pig had an IHC score of three, while the placebo groups (P-CO and P-LI) had a more intense and faster increase, with more pigs reaching a score of three on D43 (21 days post-LI challenge) (Figure 3). The IHC photos are available in the Supplementary Material (Figure S1).

### 3.4. L. intracellularis-Specific Systemic Humoral Immune Response

LI-vaccinated pigs from V-CO and V-LI showed anti-LI serum IgG production even before LI challenge and the more days post-infection, the higher the serum IgG titration, with no pig under a titration of 120 on 21 days post-LI infection (ranging from 120 to 480). After LI challenge, anti- LI serum IgG levels were detected earlier and at higher levels on LI-vaccinated pigs (V-LI and V-CO) compared to the placebo groups (P-LI and P-CO)—a booster effect (Figure 3). For the placebo groups (P-LI and P-CO), serum IgG was only identified on 14 and/or 21 days post LI infection. IgG titers of P-LI and P-CO were also lower when compared to vaccinated groups (V-LI and V-CO), with results ranging from 0 to 240.

### 3.5. Antigen-Specific Cell-Mediated Immune Response (ELISPOT Assay)

#### 3.5.1. Systemic Response (PBMC Samples)

The average number of spots per well in unstimulated (no antigen) and McCoy cell-stimulated wells was 5.95 (StD = 2.66) and 6.19 (StD = 3.6), respectively. Thus, we established a threshold of seven spots per well to consider unspecific cell-mediated immune response. ConA-stimulated wells had an average of 112.71 (StD = 33.32) spots per well.

**Figure 4:**
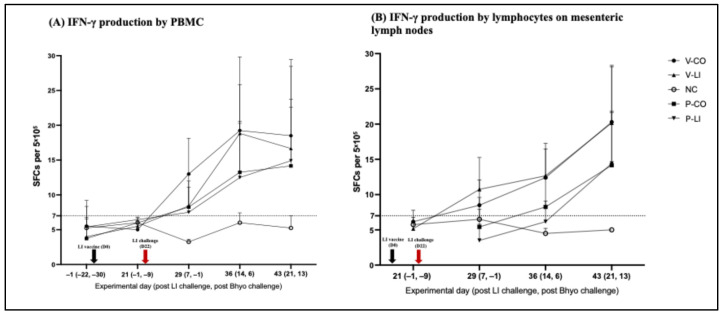
LI-specific IFN-γ production. (**A**) IFN-γ production by PBMCs, showing the systemic cell-mediated immune response. (**B**) IFN-γ production by lymphocytes on mesenteric lymph nodes, showing the gut-associated cell-mediated immune response. The results are expressed in spots-forming cells (SFCs) per 5 × 10^5^ cells (total number of cells in each well of the ELISPOT 96-well plate). Each spot represents the average number of spots associated with each individual pig in each group in each time point. The bars represent the standard deviation.

After LI challenge, the cell-mediated systemic immune response increased in the LI-vaccinated (V-CO and V-LI) and placebo (P-CO and P-LI) groups. IFN-γ production in V-CO and V-LI reached the peak of production on D36—14 days after challenge (Figure 4)—and had higher IFN-γ production compared to P-CO and P-LI. From D29 to D43, 22 out of 36 of the LI-vaccinated pigs (V-CO+V-LI) had an LI-specific IFN-γ response above the threshold for unspecific response vs. 19 out of 30 of the placebo pigs (P-CO+P-LI).

#### 3.5.2. Gut-Associated Response (LN Samples)

The average number of spots per well in unstimulated (no antigen) and McCoy cell-stimulated were 4.65 (StD = 3.1) and 6.55 (StD = 3.55), respectively. The threshold for unspecific IFN-γ response was also seven spots per well. ConA-stimulated wells had an average of 125.25 (StD = 36.72) spots per well.

Despite that only numerical differences were found between groups, gut-associated cell-mediated immune response was detected earlier in the LI-vaccinated groups, starting on D29 (7 days post-LI infection) and reached the peak at D43 (21 days post-LI infection). A total of 28 out of 35 pigs of the LI-vaccinated groups (V-CO+V-LI) vs. 15 out of 30 pigs of the placebo groups (P-CO+P-LI) had an IFN-γ response above the threshold for an unspecific response (Figure 4).

### 3.6. Non-Specific Cell-Mediated Immune Response (Flow Cytometry)

#### 3.6.1. Systemic Response (PBMC Samples)

Flow cytometry was performed on PBMCs to evaluate changes in the frequency of systemic immune cell phenotypes. On experimental day 43 (21 days post-LI challenge/13 days post-Bhyo challenge), T CD3+ cells decreased in all treatment groups compared to the negative control group. Conversely, although not statistically significant, the frequencies of CD3+ T cell subsets, T CD8+ (CD3+CD4-CD8+), and double-positive T cells (CD3+CD4+CD8+), increased in all treatment groups. Only P-CO pigs experienced a significant increase in the frequency of double-positive T cells (CD3+CD4+CD8+) on D43 compared to the NC group.

The placebo groups (P-CO and P-LI) on D36 and D43 showed higher frequencies of granulocytes compared to the LI-vaccinated groups (V-CO and V-LI) and the negative control group (NC), even though the differences were not statistically significant. The plots showing the quantification of immune cells in PBMCs are provided in the Supplementary Material (Figure S2).

#### 3.6.2. Gut-Associated Response (LN, IEL, and PP Samples)

Flow cytometry was also performed on three gut-related tissues—mesenteric lymph nodes (LNs), Peyer’s patches (PPs), and intestinal fragments—to assess intraepithelial lymphocytes (IEL) to evaluate the local frequencies of immune cells.

On LNs, seven days post-LI challenge, the CD3+ T cell frequency increased in two out of the four (V-CO and P-CO) experimental groups compared to the NC. However, on the following days, CD3+ T cell frequencies in all vaccinated/challenged groups did not differ from the NC. When analyzing CD3+ T cell subsets, no significant changes were detected in T helper (CD3+CD4+CD8-) frequencies, but on D36, T CD8+ (CD3+CD4-CD8+) and T regulatory (CD4+FoxP3+) frequencies statistically decreased in all treatment groups (V-CO, P-CO, V-LI, and P-LI) and in V-CO, respectively, compared to the NC. Although not significant, frequencies of T regulatory cells also decreased in P-CO and V-LI on D36.

On PPs, the experimental group V-CO experienced a significant decrease in the CD4+ T cell population on D43. V-LI and P-LI also experienced reductions, although they were not significant. A decrease in CD8+ T cell frequencies was also observed on D36 and D43 in all treatment groups (V-CO, P-CO, V-LI, and P-LI) compared to the NC group.

On IEL, V-CO and V-LI experienced increased CD3+ T cells on D29 and D36 compared to the NC, but this shifted on D43 when those groups had decreased CD3+ T cell frequencies compared to the NC. P-LI had an increased CD4+ T cell frequency on D29 compared to the NC group, but it returned to lower levels, similar to the NC group, on the following days. On the other hand, CD8+ T cell frequencies were significantly lower on D36 compared to the NC group. Although not significant, the frequencies of TCD8+ cells remained low compared to the control group (NC) on D43. Interestingly, V-CO, P-CO, and V-LI pigs had increased B cell frequencies on D43 in the intestine compared to the NC—even though the differences were not significant. Both placebo groups (P-CO and P-LI) had increased frequencies of granulocytes on D43 compared to the NC group. The plots showing the quantification of immune cells in LNs, PPs, and IEL are provided in the Supplementary Material (Figure S3).

## 4. Discussion

Understanding the local and systemic immune response against LI is the basis for developing better control measures to mitigate the economic impact of PE. In the present study, we characterized the post-vaccination humoral and cell-mediated immune (CMI) responses in pigs immunized against LI and co-infected with LI and Bhyo, aiming to develop a better understanding about the impact of co-infections on the immune responses induced by the LI vaccine. Bhyo was specifically chosen given the increasing prevalence of this pathogen over the last decade after regulatory changes restricted antimicrobial use in food-producing animals [29,30,31].

To our knowledge, this is the first study to analyze the impact of LI + Bhyo co-infection on the post-vaccination immune response. Also, we provided, for the first time, a detailed CMI response induced by LI in four distinct tissues (blood, mesenteric lymph nodes, Peyer’s patches, and intestinal wall). This allows for a deep understanding of the immune system landscape against LI.

In two time points (D31 and D36) LI fecal shedding was higher in the placebo co-infected group compared to the LI-vaccinated and placebo-infected with LI (P-CO > V-LI and P-LI) groups. A previous study conducted using the same killed IM vaccine reported a 15-fold reduction in the mean daily average amount of LI fecal shedding [6]. Another study has analyzed a live IM LI vaccine and observed a reduction on LI fecal shedding only 14 days post-infection [18]. It reinforces the intermittent characteristic of LI shedding, and it shows that Bhyo co-infection may increase LI fecal shedding when pigs are not vaccinated.

LI vaccine was effective in the reduction in intestinal colonization, even in animals co-infected with Bhyo. While LI-vaccinated pigs kept a lower average IHC score throughout the time, the placebo pigs gradually increased the IHC’s average score. At 21 days post-infection, also known as the peak of the disease [32], all pigs in the placebo groups were positive and 50% (6/12) of them had the highest level of intestinal LI colonization.

Since the vaccine used in the present study was intramuscularly administered, it was expected to observe increased serological anti-LI IgG compared to placebo animals, as observed in killed [6], live [18], and subunit [32] IM vaccines. However, this is the first study where 100% of the LI-vaccinated pigs produced LI serum IgG at 14 and 21 days post-LI challenge. Also, this is the first study to analyze the humoral responses in pigs LI-vaccinated and co-infected with LI+Bhyo. We observed that LI+Bhyo co-infection did not affect the post-vaccination systemic humoral immune response.

Previous studies have shown the ability of LI to modulate the immune system. Macintyre et al. (2003) [16] described a correlation between the presence of LI in the gut and reduced local TCD8+ and B cells; Jacobson et al. (2011) [33] reported limited expression of cytokines in both sera and intestines, indicating downregulation of the immune response. Both studies were performed with pigs exposed to LI antigens for the first time. Here, we show that even when pigs were previously exposed to LI vaccine antigens and then challenged, LI downregulates local TCD4+ and TCD8+ responses. Additionally, co-infected pigs demonstrated more intense downregulation of TCD8+ cells (in PPs and IEL).

On the other hand, results show that the systemic CMI response may play an important role in LI infection regardless of co-infection or previous exposure to the vaccine antigen. T CD8+ and double-positive memory T cell (CD4+CD8+) frequencies increased in PBMCs compared to negative control (NC) pigs. These results corroborate the findings reported by Cordes et al. (2012) [19], in which it was identified that IFN-γ was mainly secreted by CD4-CD8+ and double-positive memory CD4+CD8+ T cells isolated from blood.

The results from the present study reinforce the role of immunoglobulins in the local immune response. LI-vaccinated groups had higher B cell frequencies compared to P-LI and the NC at 21 days post-LI challenge. Those B cells are assumed to secrete specific LI and/or Bhyo immunoglobulins in the intestinal mucosa, as previous studies have demonstrated [15,34]. This finding suggest that the IM vaccination is likely capable to induce the production of intestinal immunoglobulins and that this immune pathway is important for controlling LI infection.

In addition, since T regulatory cells (CD4+FoxP3+) are known for their role in controlling the immune response, including the suppression of T helper CD4+ and T CD8+ cells [35], we hypothesized that T reg cells could be important in the mechanism used by LI for immunomodulation. At 7 days post-LI challenge T reg frequencies were equal to or higher than the negative control group. Nonetheless, these frequencies shifted to equal or lower at 14 and 2 post-LI challenge. Further studies are needed to understand the ability of LI to downregulate the immune system. The reduced number of animals per time point is an important limitation of the present study. But comprehensive immunological assessments include time-sensitive procedures that limit the ability to work with a large number of samples. These limitations were also observed in other studies that used similar methodologies [36,37,38].

## 5. Conclusions

The immune response induced by an IM LI vaccine was not affected by LI+Bhyo co-infection. LI-vaccinated co-infected pigs showed reduced LI intestinal colonization and intestinal gross. There were no significant changes in IFN-γ when pigs were vaccinated and co-infected with Bhyo or LI. B cells seem to play an important role in the local immune response, and T regulatory cells apparently do not have a significant role in the mechanisms used by LI for immunomodulation. This study contributes to a better understanding of LI immune response and can provide subtract for further research in the control of LI.

## Figures and Tables

**Table 1:** List of antibodies used in flow cytometry.

Cell Marker	Antibody (Brand)	Isotype Control Antibody (Brand)
CD3	CD3-PE-Cy7 (BD Bioscience, San Jose, CA, USA)	PE/Cyanine 7 Mouse IgG2a (Biolegend, San Diego, CA, USA)
CD4	CD4-PerCP-Cy5.5 (BD Bioscience, San Jose, CA, USA)	PerCP-Cy5.5 Mouse IgG2b (Biolegend, San Diego, CA, USA)
CD8	CD8a-FITC (BD Bioscience, San Jose, CA, USA)	FITC Mouse IgG2a (Biolegend, San Diego, CA, USA)
CD21	CD21 APC (BD Bioscience, San Jose, CA, USA)	APC Mouse IgG1 (Biolegend, San Diego, CA, USA)
CD172a	PE Mouse Anti-pig (BD Bioscience, San Jose, CA, USA)	PE Mouse IgG2b (Biolegend, San Diego, CA, USA)
CD79	CD79-APC eFlour 780 (BD Bioscience, San Jose, CA, USA)	APC/Cyanine 7 Mouse IgG1(Biolegend, San Diego, CA, USA)
FOXP3	FOXP3 PE (BD Bioscience, San Jose, CA, USA)	Rat IgG2a (Invitrogen, Waltham, MA, USA)

**Table 2:** PCR CT values of colonic intestinal content of necropsied pigs after Bhyo challenge.

		CT Value—*B. hyodysentariae*	
	Animal ID	Day 36	Day 43
V-CO	3	neg	-
	10	neg	-
	14	neg	-
	15	neg	-
	20	neg	-
	24	26.3	-
	6	-	35.74
	16	-	neg
	31	-	neg
	48	-	neg
	56	-	36.54
	75	-	neg
P-CO	18	29.7	-
	23	neg	-
	40	neg	-
	65	33.21	-
	77	35.38	-
	79	neg	-
	4	-	32.55
	8	-	neg
	27	-	30.11
	58	-	neg
	73	-	neg
	80	-	neg

CT value = cycle threshold value; V-CO = LI-vaccinated and co-infected with LI and Bhyo group; P-CO = placebo and co-infected with LI and Bhyo group; neg = negative.

**Table 3:** *L. intracellularis* fecal shedding throughout time.

	Experimental Day (Days Post-LI Challenge, Days Post-Bhyo Challenge)															
	27 (5, −3)		29 (7, −1)		31 (9, 1) *		34 (12, 3)		36 (14, 6) *		38 (16, 8)		41 (19, 11)		43 (21, 13)	
	Pigs Shedding/Pigs Tested (%)	Average Ct Value (StD)	Pigs Shedding/Pigs Tested (%)	Average Ct Value (StD)	Pigs Shedding/Pigs Tested (%)	Average Ct Value (StD)	Pigs Shedding/Pigs Tested (%)	Average Ct Value (StD)	Pigs Shedding/Pigs Tested (%)	Average Ct Value (StD)	Pigs Shedding/Pigs Tested (%)	Average Ct Value (StD)	Pigs Shedding/Pigs Tested (%)	Average Ct Value (StD)	Pigs Shedding/Pigs Tested (%)	Average Ct Value (StD)
V-CO	12/18 (66.6%)	36.24 (3.72)	17/18 (94.4%)	31.44 (3.67)	12/12 (100%)	30.07 (2.65) ^ab^	11/12 (91.6%)	31.59 (4.75)	10/11 (90.9%)	31.33 (4.70) ^ab^	2/6 (33.3%)	36.89 (4.84)	3/6 (50%)	35.78 (5.56)	5/6 (83.3%)	33.90 (5.82)
P-CO	16/18 (88.8%)	33.23 (3.55)	11/12 (91.6%)	31.06 (5.16)	12/12 (100%)	27.67 (3.45) ^a^	12/12 (100%)	27.29 (4.61)	12/12 (100%)	27.44 (4.67) ^a^	6/6 (100%)	33.77 (2.56)	6/6 (100%)	32.62 (4.42)	6/6 (100%)	32.14 (4.04)
V-LI	13/18 (72.2%)	36.3 (2.81)	16/18 (88.8%)	33.87 (3.69)	10/12 (83.3%)	32.66 (5.12) ^b^	10/12 (83.3%)	31.82 (4.58)	7/8 (87.5%)	33.70 (3.49) ^b^	5/6 (83.3%)	34.94 (4.00)	4/6 (66.6%)	36.12 (3.75)	6/6 (100%)	35.45 (1.73)
P-LI	7/10 (70%)	33.92 (4.86)	7/9 (77.7%)	34.7 (3.78)	8/9 (88.8%)	32.26 (5.12) ^b^	9/9 (100%)	32.20 (5.15)	7/9 (77.7%)	33.60 (4.86) ^b^	4/6 (66.6%)	30.91 (7.30)	6/6 (100%)	29.44 (5.74)	5/6 (83.3%)	30.74 (5.67)

Ct value = cycle threshold value; StD = standard deviation; V-CO = LI-vaccinated and co-infected with LI + Bhyo group; P-CO = placebo and co-infected with LI + Bhyo group. Statistically significant differences between groups within an experimental day are represented as *. Different letters in the same column indicate a statistically significant difference (*p* < 0.05). Letters were not included on experimental days with no statistically significant differences.

**Table 4:** *B. hyodysenteriae* fecal shedding throughout time.

Experimental Day (Days Post-LI Challenge, Days Post-Bhyo Challenge)												
	31 (9, 1)		34 (12, 3)		36 (14, 6)		38 (16, 8)		41 (19, 11)		43 (21, 13)	
	Pigs Shedding/Pigs Tested (%)	Average Ct Value (StD)	Pigs Shedding/Pigs Tested (%)	Average Ct Value (StD)	Pigs Shedding/Pigs Tested (%)	Average Ct Value (StD)	Pigs Shedding/Pigs Tested (%)	Average Ct Value (StD)	Pigs Shedding/Pigs Tested (%)	Average Ct Value (StD)	Pigs Shedding/Pigs Tested (%)	Average Ct Value (StD)
V-CO	0/12 (0%)	40.00 (0.00)	0/12 (0%)	40.00 (0.00)	2/12 (16.66%)	39.36 (1.51)	0/6 (0%)	40.00 (0.00)	0/6 (0%)	40.00 (0.00)	0/6 (0%)	40.00 (0.00)
P-CO	0/12 (0%)	40.00 (0.00)	1/12 (8.33%)	39.55 (1.57)	4/12 (33.33%)	37.78 (3.54)	0/6 0%)	40.00 (0.00)	0/6 (0%)	40.00 (0.00)	1/6 (16.66%)	39.75 (0.62)

Ct value = cycle threshold value; StD = standard deviation; V-CO = LI-vaccinated and co-infected with LI + Bhyo group; P-CO = placebo and co-infected with LI + Bhyo group. No statistically significant differences were observed at any of the time points.

## Data Availability

The original contributions presented in this study are included in the article/Supplementary Materials. Further inquiries can be directed to the corresponding authors.

## Supplementary Materials

The following supporting information can be downloaded at: https://www.mdpi.com/article/10.3390/ani16010114/s1. Table S1: Fecal score. Table S2: PCR CT values of fecal swabs after *L. intracellularis* challenge. Table S3: Description of gross lesions found on D43 in every pig of the study groups. Figure S1: Immunohistochemistry of pigs challenged with LI. Immunostaining of LI is shown in dark brown, pointed by arrows, in crypt enterocytes in the ileum. Figure S2: Quantification of immune cells in PBMC measured by flow cytometry, showing the systemic cell mediated immune (CMI) response. Figure S3: Quantification of immune cells in (A) LN, (B) PP and (C) IEL, measured by flow cytometry, showing the gut-associated cell mediated immune (CMI) response.

## Abbreviations

The following abbreviations are used in this manuscript:

ANOVA	One-way analysis of variance
Bhyo	*Brachyspira hyodysenteriae*
CMI	Cell-mediated immune response
CON A	Concanavalin A
CT	Threshold cycles
EDTA	Ethylenediaminetetraacetic acid
ELISPOT	Enzyme-linked immuno spot
IACUC	Institutional Animal Care and Use Committee
IEL	Intraepithelial lymphocytes
IHC	Immunohistochemistry
IM	Intramuscular
IPMA	Immunoperoxidase monolayer assay
LI	*Lawsonia intracellularis*
LN	Lymph nodes
PBMC	Peripheral blood mononuclear cells
PBS	Phosphate-Buffered Saline
PCR	Polymerase chain reaction
PE	Proliferative enteropathy
PP	Peyer’s patch
SD	Swine dysentery
StD	Standard deviation
VDL-UMN	Veterinary Diagnostic Lab of the University of Minnesota
