# Supplementary material for: Immune Response of Pigs Vaccinated Against Proliferative Enteropathy and Co-Infected with Lawsonia intracellularis and Brachyspira hyodysenteriae
Source: Animals (Basel). 2025 Dec 31;16(1):114. doi: 10.3390/ani16010114 (PMC12785021; doi:10.3390/ani16010114)
Supplement: Supplementary file 1 [file animals-16-00114-s001.zip › animals-4004426-supplementary.pdf]

Supplementary material

Immune response of pigs vaccinated against proliferative enteropathy and co-infected with *Lawsonia intracellularis* and *Brachyspira hyodysenteriae*

Summary

Table S1. Fecal score 2

Table S2. PCR CT values of fecal swabs after *L. intracellularis* challenge. 5

Table S3. Description of gross lesions found on D43 in every pig of the study groups...................8

Figure S1. Immunohistochemistry of pigs challenged with LI. Immunostaining of LI is shown in dark brown, pointed by arrows, in crypt enterocytes in the ileum 10

Figure S2. Quantification of immune cells in PBMC measured by flow cytometry, showing the systemic cell mediated immune (CMI) response. 11

Figure S3. Quantification of immune cells in A) LN, B) PP and C) IEL, measured by flow cytometry, showing the gut-associated cell mediated immune (CMI) response............................13

Table S1. Fecal score

	Fecal score																		
**GROUP**	**PIG ID**	**D23**	**D24**	**D25**	**D26**	**D27**	**D28**	**D29**	**D31**	**D33**	**D34**	**D35**	**D36**	**D37**	**D38**	**D39**	**D4**	**D41**	**D42**
V-CO	1	1	5	1	1	1	1	1	NPO	NA	NA	NA	NA	NA	NA	NA	NA	NA	NA
	2	NA	NA	NA	NA	NA	NA	NA	NA	NA	NA	NA	NA	NA	NA	NA	NA	NA	NA
	3	1	1	2	1	1	1	1	NPO	NPO	2	2	NPO	4	NPO	2	2	NPO	NPO
	6	1	1	1	1	1	1	1	1	NPO	1	NPO	NPO	NA	NA	NA	NA	NA	NA
	10	1	1	NPO	1	NPO	5	1	NPO	NPO	3	NPO	NPO	NPO	1	1	2	2	2
	12	1	3	5	5	5	3	1	NPO	NA	NA	NA	NA	NA	NA	NA	NA	NA	NA
	14	1	1	3	5	1	4	1	3	3	3	3	NPO	4	4	5	3	4	4
	15	1	2	1	1	5	4	1	5	NPO	5	4	NPO	4	1	2	3	4	NPO
	16	1	1	3	1	3	4	1	1	3	5	2	NPO	NA	NA	NA	NA	NA	NA
	20	1	1	1	1	4	4	1	1	NPO	NPO	NPO	NPO	2	1	5	2	3	2
	21	1	1	4	1	1	4	1	NPO	NA	NA	NA	NA	NA	NA	NA	NA	NA	NA
	24	1	4	3	1	2	4	1	1	3	NPO	2	NPO	NPO	1	5	NPO	4	5
	26	NA	NA	NA	NA	NA	NA	NA	NA	NA	NA	NA	NA	NA	NA	NA	NA	NA	NA
	28	NA	NA	NA	NA	NA	NA	NA	NA	NA	NA	NA	NA	NA	NA	NA	NA	NA	NA
	31	1	1	5	1	NPO	4	1	NPO	4	3	3	NPO	NA	NA	NA	NA	NA	NA
	48	1	3	3	1	NPO	4	1	1	NPO	NPO	4	NPO	NA	NA	NA	NA	NA	NA
	49	1	3	2	1	5	4	1	NPO	NA	NA	NA	NA	NA	NA	NA	NA	NA	NA
	56	1	3	1	1	5	4	1	2	NPO	4	NPO	NPO	NA	NA	NA	NA	NA	NA
	66	1	1	1	1	1	1	1	NPO	NA	NA	NA	NA	NA	NA	NA	NA	NA	NA
	68	1	3	3	1	5	4	1	NPO	NA	NA	NA	NA	NA	NA	NA	NA	NA	NA

	75	4	2	4	5	1	1	4	NPO	3	NPO	NPO	NPO	NA	NA	NA	NA	NA	NA
P-CO	4	5	2	5	NPO	1	5	3	3	3	NPO	NPO	NPO	5	5	5	4	5	4
	7	1	1	1	NPO	2	4	3	NPO	NA	NA	NA	NA	NA	NA	NA	NA	NA	NA
	8	1	2	1	NPO	1	1	1	2	NPO	4	1	NPO	5	NPO	1	NPO	NPO	2
	17	1	3	5	NPO	3	4	1	NPO	NA	NA	NA	NA	NA	NA	NA	NA	NA	NA
	18	1	1	1	NPO	NPO	1	1	1	NPO	NPO	NPO	NPO	NA	NA	NA	NA	NA	NA
	23	1	1	3	NPO	1	1	2	1	NPO	NPO	NPO	NPO	NA	NA	NA	NA	NA	NA
	27	1	1	1	3	3	4	3	3	NPO	2	NPO	NPO	NPO	5	5	4	NPO	5
	40	1	1	3	NPO	1	1	1	4	3	2	NPO	NPO	NA	NA	NA	NA	NA	NA
	43	5	5	1	NPO	2	2	1	NPO	NA	NA	NA	NA	NA	NA	NA	NA	NA	NA
	44	1	3	2	NPO	1	1	3	NPO	NA	NA	NA	NA	NA	NA	NA	NA	NA	NA
	47	1	1	2	NPO	5	3	4	NPO	NA	NA	NA	NA	NA	NA	NA	NA	NA	NA
	58	1	4	1	NPO	1	1	1	NPO	NPO	4	2	NPO	NPO	NPO	NPO	1	1	2
	65	1	2	1	NPO	1	3	5	5	NPO	4	NPO	NPO	NA	NA	NA	NA	NA	NA
	71	1	1	1	NPO	1	3	3	NPO	NA	NA	NA	NA	NA	NA	NA	NA	NA	NA
	73	2	5	1	NPO	2	1	2	5	4	NPO	3	NPO	NPO	3	4	3	3	3
	77	1	1	1	NPO	1	1	3	NPO	3	4	NPO	NPO	NA	NA	NA	NA	NA	NA
	79	1	2	1	NPO	1	1	1	3	NPO	NPO	NPO	NPO	NA	NA	NA	NA	NA	NA
	80	1	5	3	NPO	1	3	1	3	3	5	NPO	NPO	5	5	5	5	5	5
V-LI	5	1	1	1	1	1	NPO	2	2	4	2	NPO	NPO	NA	NA	NA	NA	NA	NA
	13	1	1	3	1	1	1	1	2	NPO	4	NPO	NPO	5	5	4	4	1	NPO
	19	1	1	1	1	NPO	1	1	2	NPO	4	4	NPO	NPO	4	NPO	4	NPO	NPO
	22	1	1	1	1	1	1	1	NPO	3	1	NPO	NPO	NA	NA	NA	NA	NA	NA
	30	1	2	3	1	NPO	5	1	NPO	NA	NA	NA	NA	NA	NA	NA	NA	NA	NA
	32	1	1	3	1	1	5	1	NPO	NA	NA	NA	NA	NA	NA	NA	NA	NA	NA

	33	1	1	1	1	2	1	1	1	NPO	2	NPO	NPO	NA	NA	NA	NA	NA	NA
	36	1	3	1	4	NPO	1	1	NPO	NA	NA	NA	NA	NA	NA	NA	NA	NA	NA
	37	1	1	1	1	5	1	1	NPO	NPO	NPO	NPO	NPO	NPO	2	NPO	3	2	2
	39	1	1	1	1	1	1	1	1	NPO	4	NPO	NPO	NPO	1	NPO	NPO	4	NPO
	42	1	1	1	1	1	1	1	NPO	NA	NA	NA	NA	NA	NA	NA	NA	NA	NA
	50	1	1	1	1	NPO	1	3	1	NPO	1	NPO	NPO	NPO	1	NPO	2	1	2
	51	1	2	1	1	NPO	4	1	1	3	NPO	NPO	NPO	NA	NA	NA	NA	NA	NA
	52	1	2	1	1	1	1	1	NPO	NA	NA	NA	NA	NA	NA	NA	NA	NA	NA
	53	NA	NA	NA	NA	NA	NA	NA	NA	NA	NA	NA	NA	NA	NA	NA	NA	NA	NA
	60	1	1	1	1	NPO	3	1	NPO	NA	NA	NA	NA	NA	NA	NA	NA	NA	NA
	63	1	1	1	1	1	1	1	1	NPO	2	NPO	NPO	NPO	NPO	2	1	1	2
	64	NA	NA	NA	NA	NA	NA	NA	NA	NA	NA	NA	NA	NA	NA	NA	NA	NA	NA
	72	1	1	1	1	1	4	2	1	NPO	3	1	NPO	NA	NA	NA	NA	NA	NA
	74	1	1	1	1	1	2	1	3	NPO	1	NPO	NPO	NA	NA	NA	NA	NA	NA
	78	NA	NA	NA	NA	NA	NA	NA	NA	NA	NA	NA	NA	NA	NA	NA	NA	NA	NA
P-LI	25	3	1	1	1	1	2	1	2	NPO	4	NPO	NPO	NPO	5	2	5	5	5
	29	1	1	1	1	1	1	1	2	NPO	2	NPO	NPO	NPO	1	NPO	1	1	1
	38	1	1	1	1	1	2	1	2	NPO	1	NPO	NPO	4	5	5	5	5	4
	45	1	1	3	1	3	4	1	3	3	4	1	NPO	NA	NA	NA	NA	NA	NA
	46	1	1	1	1	NPO	1	1	1	3	1	NPO	NPO	4	5	NPO	4	4	NPO
	54	1	1	3	1	1	2	1	NPO	NA	NA	NA	NA	NA	NA	NA	NA	NA	NA
	57	2	1	2	1	NPO	3	1	5	3	4	NPO	NPO	NA	NA	NA	NA	NA	NA
	61	1	1	1	1	4	3	1	1	NPO	1	NPO	NPO	NPO	3	NPO	NPO	NPO	NPO
	62	1	1	1	1	NPO	1	1	NPO	NA	NA	NA	NA	NA	NA	NA	NA	NA	NA
	67	1	1	1	1	NPO	1	1	3	NPO	1	1	NPO	NA	NA	NA	NA	NA	NA

	69	1	1	1	1	1	1	1	NPO	NA	NA	NA	NA	NA	NA	NA	NA	NA	NA
	70	1	1	2	1	1	1	1	4	NPO	2	NPO	NPO	NPO	1	1	2	1	2
NC	9	1	1	1	1	1	1	1	1	1	1	1	1	1	1	1	1	1	1
	11	NA	NA	NA	NA	NA	NA	NA	NA	NA	NA	NA	NA	NA	NA	NA	NA	NA	NA
	34	1	1	1	1	1	1	1	1	1	1	1	1	NA	NA	NA	NA	NA	NA
	35	1	1	1	3	1	1	1	1	1	2	1	1	1	1	1	1	1	1
	41	NA	NA	NA	NA	NA	NA	NA	NA	NA	NA	NA	NA	NA	NA	NA	NA	NA	NA
	55	1	1	1	1	2	1	1	NPO	NA	NA	NA	NA	NA	NA	NA	NA	NA	NA
	59	1	1	1	1	1	1	1	1	1	1	1	1	NA	NA	NA	NA	NA	NA
	76	1	1	1	1	1	1	1	NPO	NA	NA	NA	NA	NA	NA	NA	NA	NA	NA

V-CO = LI vaccinated and co-infected with LI+Bhyo, P-CO = placebo vaccinated and co-infected with LI+Bhyo, V-LI = LI vaccinated and infected with LI, P-LI = placebo vaccinated and infected with LI, NC = negative control, placebo vaccinated and non-challenged; NPO = not possible to observe; NA = NA = non applicable (euthanized pig).

Table S2. PCR CT values of fecal swabs after *L. intracellularis* challenge.

	CT value - *L. intracellularis*								
	Pig ID	Day 27	Day 29	Day 31	Day 34	Day 36	Day 38	Day 41	Day 43
V-CO	1	32.84	29.08	NA	NA	NA	NA	NA	NA
	2	NA	NA	NA	NA	NA	NA	NA	NA
	3	34.12	31.91	31.07	35.52	35.88	neg	neg	38.94
	6	neg	33.24	32.12	31.4	32.11	NA	NA	NA
	10	31.83	33.51	27.32	26.56	ERROR	neg	37.07	34.5
	12	neg	38.04	NA	NA	NA	NA	NA	NA
	14	32.46	28.9	34.16	34.99	34.33	neg	neg	36.13
	15	31.96	29.96	29.47	neg	36.15	neg	neg	neg
	16	33.04	30.48	29.75	24.79	25.04	NA	NA	NA

	20	30.57	33.23	26.86	30.41	26.75	31.31	27.69	28.42
	21	neg	30.03	NA	NA	NA	NA	NA	NA
	24	30.87	24.09	24.78	30.93	29.83	30	29.93	25.43
	26	NA	NA	NA	NA	NA	NA	NA	NA
	28	NA	NA	NA	NA	NA	NA	NA	NA
	31	neg	32.48	31.7	26.49	28.04	NA	NA	NA
	48	36.37	30.15	30.69	34.16	28.18	NA	NA	NA
	49	neg	31.93	NA	NA	NA	NA	NA	NA
	56	31.78	32.77	30.27	27.16	28.28	NA	NA	NA
	66	neg	neg	NA	NA	NA	NA	NA	NA
	68	33.32	26.85	NA	NA	NA	NA	NA	NA
	75	35.24	29.26	32.59	36.64	neg	NA	NA	NA
P-CO	4	36.37	26.67	28.52	32.31	31.81	32.53	36.74	32.4
	7	32.8	NA	NA	NA	NA	NA	NA	NA
	8	30.8	29.61	26.13	25.19	32.36	36.01	35.59	35.11
	17	30.03	NA	NA	NA	NA	NA	NA	NA
	18	31.73	27.23	26.4	26.54	23.04	NA	NA	NA
	23	neg	32.56	24.28	29.87	25.54	NA	NA	NA
	27	30.83	28.49	26.38	22.33	22.78	31.86	31.92	28.91
	40	32.48	neg	26.47	27.54	24.68	NA	NA	NA
	43	29.87	NA	NA	NA	NA	NA	NA	NA
	44	31.31	NA	NA	NA	NA	NA	NA	NA
	47	30.78	NA	NA	NA	NA	NA	NA	NA
	58	34.46	31.21	32.65	34.14	31.62	36.01	32.31	33.72
	65	36.32	26.78	22.69	20.48	20.53	NA	NA	NA
	71	36.4	NA	NA	NA	NA	NA	NA	NA
	73	31.46	32.84	29.96	27.01	34.52	36.01	34.7	36.76
	77	neg	37.62	29.07	26.68	24.12	NA	NA	NA
	79	35.4	36.96	34.57	33.94	26.7	NA	NA	NA
	80	27.07	22.75	24.97	21.43	31.62	30.22	24.43	25.91

V-LI	5	34.19	neg	35.42	27.15	30.07	NA	NA	NA
	13	35.43	30.31	neg	25.87	29.3	33.24	36.56	36.13
	19	neg	37.6	31.42	31.05	33.3	neg	30.12	32.4
	22	34.41	33.82	29.98	29.35	25.55	NA	NA	NA
	30	31.73	31.29	NA	NA	NA	NA	NA	NA
	32	37.13	33.36	NA	NA	NA	NA	NA	NA
	33	36.44	neg	30.39	28.46	33.55	NA	NA	NA
	36	36	36.6	NA	NA	NA	NA	NA	NA
	37	33.94	36.49	33.89	31.67	29.38	28.15	neg	36.59
	39	neg	37.54	neg	neg	neg	35.04	35.97	34.87
	42	neg	30.18	NA	NA	29.84	NA	NA	NA
	50	neg	30.75	36.83	35.48	ERROR	36.66	neg	35.37
	51	34.79	31.02	30.75	neg	33.77	NA	NA	NA
	52	36.46	29.1	NA	NA	NA	NA	NA	NA
	53	NA	NA	NA	NA	NA	NA	NA	NA
	60	35.58	36.49	NA	NA	NA	NA	NA	NA
	63	neg	35.28	31.3	32.89	32.09	36.55	34.08	37.34
	64	NA	NA	NA	NA	NA	NA	NA	NA
	72	36.28	30.67	30.72	29.39	36.76	NA	NA	NA
	74	31.03	29.12	21.18	30.57	34.22	NA	NA	NA
	78	NA	NA	NA	NA	NA	NA	NA	NA
P-LI	25	29.17	29.89	30.55	21.58	27.7	26.75	20.14	25.41
	29	ERROR	30.29	33.31	36.92	32.33	neg	34.51	31.61
	38	34.79	34.33	34.78	27.09	28.12	27.63	27.95	27.4
	45	28.65	neg	28.66	34.88	neg	NA	NA	NA
	46	30.13	32.66	30.83	30.74	35.43	22.71	28.72	25.89
	54	neg	NA	NA	NA	NA	NA	NA	NA
	57	35.16	37.61	27.27	32.38	34.01	NA	NA	NA
	61	33.24	34.55	33.57	37.69	28.24	28.36	36.5	neg
	62	neg	NA	NA	NA	NA	NA	NA	NA

	67	neg	32.93	31.36	32.85	36.53	NA	NA	NA
	69	28.07	NA	NA	NA	NA	NA	NA	NA
	70	neg	neg	neg	35.63	neg	neg	28.84	34.12

CT value = cycle threshold value; V-CO = LI vaccinated and co-infected with LI+Bhyo, P-CO = placebo vaccinated and co-infected with LI+Bhyo, V-LI = LI vaccinated and infected with LI, P-LI = placebo vaccinated and infected with LI, NC = negative control, placebo vaccinated and non-challenged; neg = negative; ERROR = sample that could not be processed; NA = non applicable (euthanized pig).

Table S3. Description of gross lesions found on D43 in every pig of the study groups.

		**Ileum gross lesions**	**Colon gross lesions**
V-CO	Pig 24	No lesions	thickening of the mucosa and excessive mucus content
	Pig 3, 10, 14, 15, 20	No lesions	No lesions
V-LI	Pig 13, 19, 37, 39, 50, 63	No lesions	No lesions
P-CO	Pig 27	edema, hyperemia, thickening of mucosa	hyperemia
	Pig 80	hyperemia, severe thickening of mucosa	hyperemia, severe thickening of the mucosa, attached intestinal content, pseudo membrane
	Pig 4	hyperemia, mild thickening of mucosa	hyperemia, thickening of the mucosa, attached intestinal content
	Pig 8, 58, 73	No lesions	No lesions
P-LI	Pig 25	severe thickening of the mucosa, multifocal necrosis of the epithelium	hyperemia

	Pig 38	mild thickening of the mucosa	No lesions
	Pig 29, 46, 61, 70	No lesions	No lesions

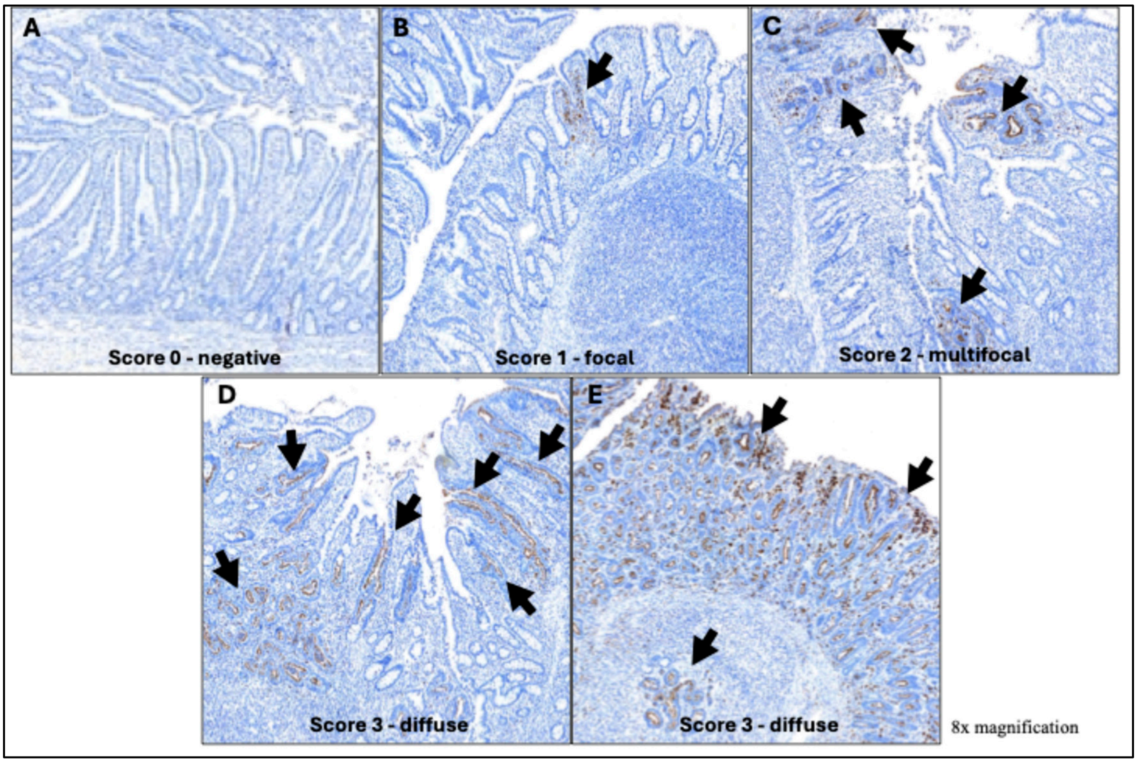

Figure S1. Immunohistochemistry of pigs challenged with LI. Immunostaining of LI is shown in dark brown, pointed by arrows, in crypt enterocytes in the ileum. A) Negative animal, with no LI immunostaining – Score 0. B) Focal presence of LI – Score 1. C) Multifocal presence of LI – Score 2. D and E) Multifocal presence of LI – Score 3.

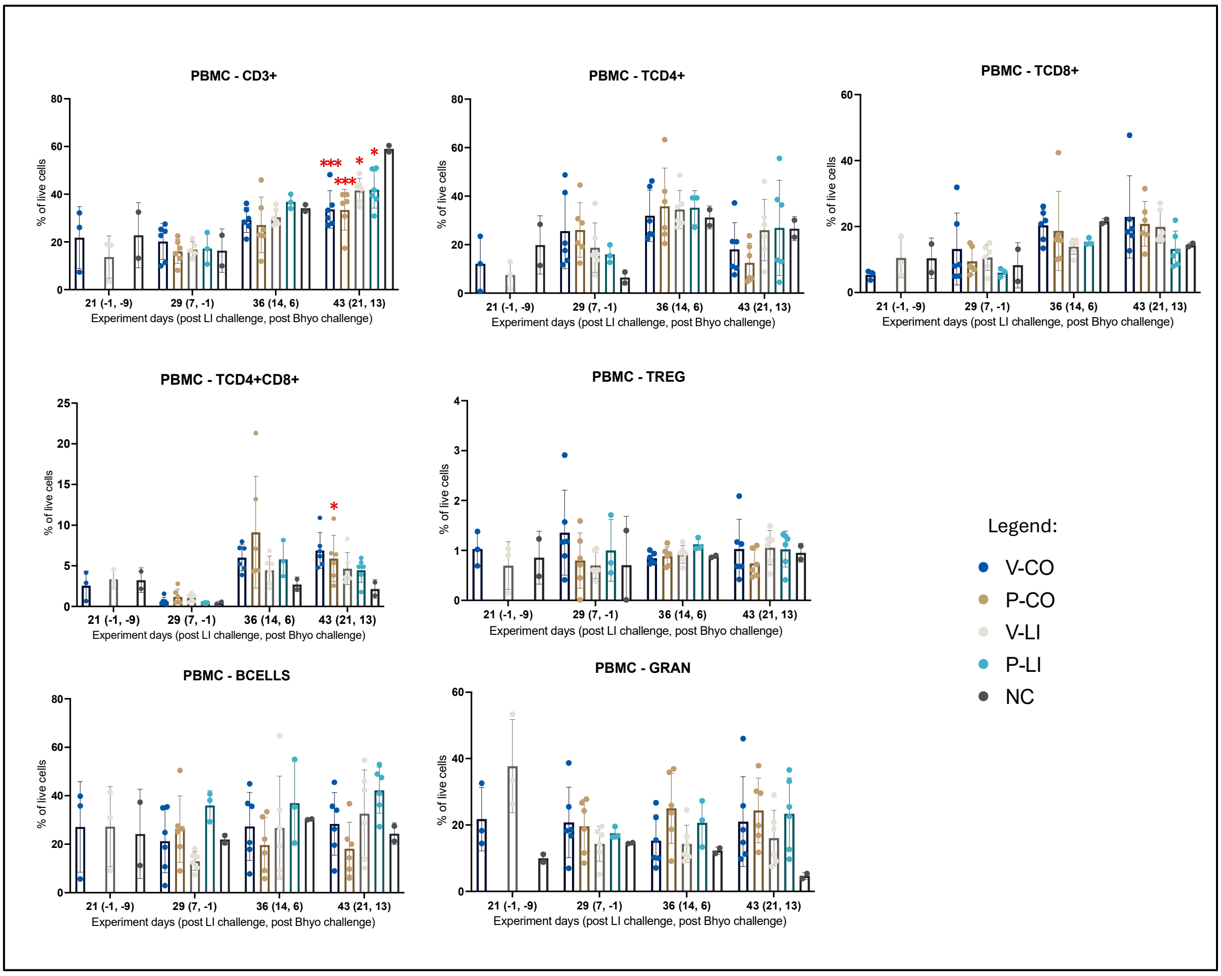

Figure S2. Quantification of immune cells in PBMC measured by flow cytometry, showing the systemic cell mediated immune (CMI) response. Each dot represents one animal. Significant differences are represented as p<0.05 (*), p<0.005 (**) and p<0.0005 (***) and show differences between treatment groups (V-CO, P-CO, V-LI, P-LI) and the negative control group (NC). PBMC = peripheral blood mononuclear cells, V-CO = LI vaccinated and co-infected with LI+Bhyo, P-CO = placebo vaccinated and co-infected with LI+Bhyo, V-LI = LI vaccinated and infected with LI, P-LI = placebo vaccinated and infected with LI, NC = negative control, placebo vaccinated and non-challenged.

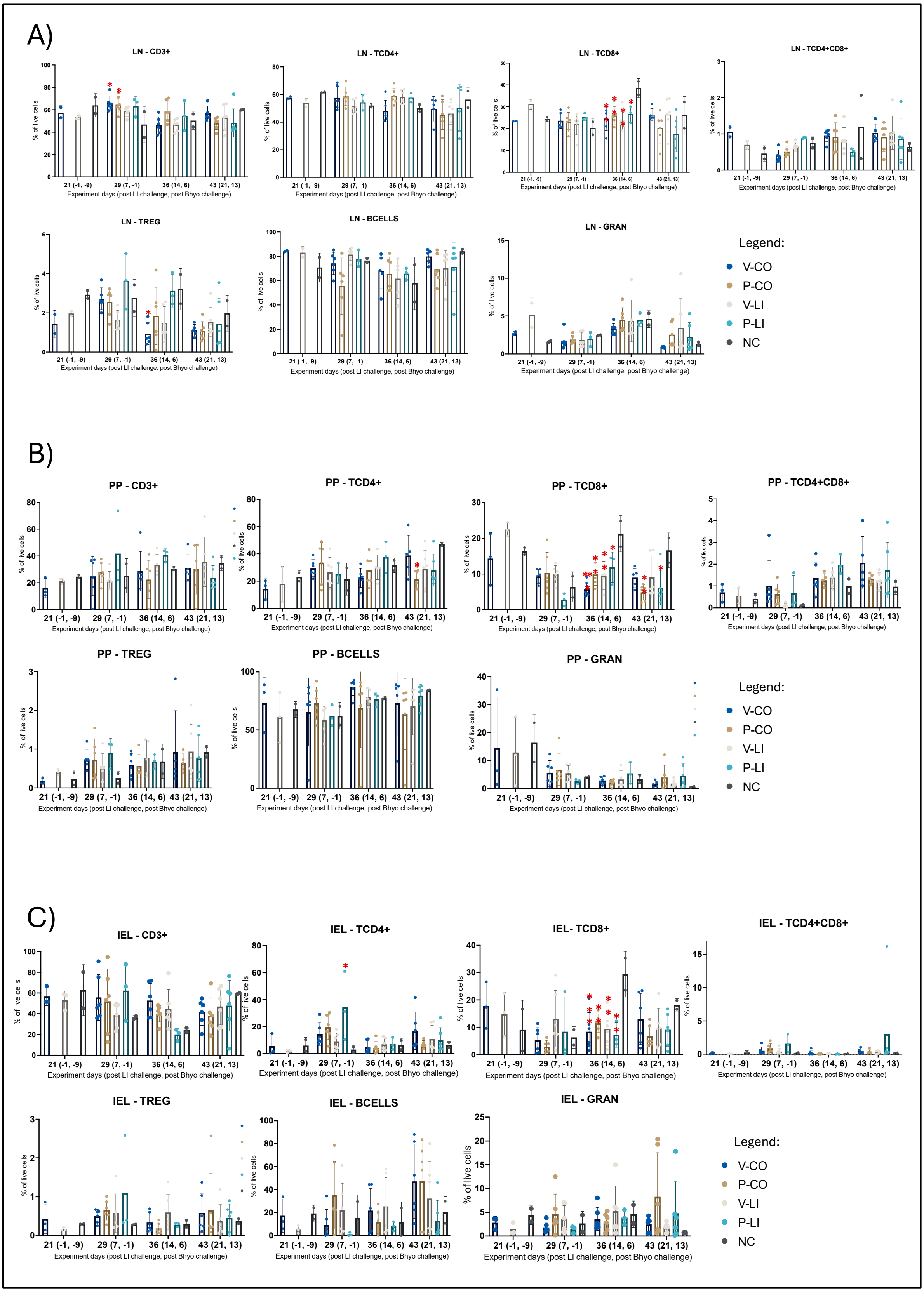
A)
LN - CD3+
LN - TCD4+
LN - TCD8+
LN - TCD4+CD8+
LN - TREG
LN - BCELLS
LN - GRAN
B)
PP - CD3+
PP - TCD4+
PP - TCD8+
PP - TCD4+CD8+
PP - TREG
PP - BCELLS
PP - GRAN
C)
IEL - CD3+
IEL - TCD4+
IEL- TCD8+
IEL - TCD4+CD8+
IEL - TREG
IEL - BCELLS
IEL - GRAN
% of live cells
21 (-1, -9)
29 (7, -1)
36 (14, 6)
43 (21, 13)
Experiment days (post LI challenge, post Bhyo challenge)
Legend:
V-CO
P-CO
V-LI
P-LI
NC

Figure S3. Quantification of immune cells in A) LN, B) PP and C) IEL, measured by flow cytometry, showing the gut-associated cell mediated immune (CMI) response. Each dot represents one animal. Significant differences are represented as $p<0.05$ (*), $p<0.005$ (**) and $p<0.0005$ (***) and show differences between treatment groups (V-CO, P-CO, V-LI, P-LI) and the negative control group (NC). LN = mesenteric lymph nodes, PP = Peyer's patches, IEL = intraepithelial lymphocytes, V-CO = LI vaccinated and co-infected with LI+Bhyo, P-CO = placebo vaccinated and co-infected with LI+Bhyo, V-LI = LI vaccinated and infected with LI, P-LI = placebo vaccinated and infected with LI, NC = negative control, placebo vaccinated and non-challenged.
